# Perception of Online Learning Among Undergraduate Medical Students of Eastern India: A Cross-Sectional Study

**DOI:** 10.7759/cureus.32580

**Published:** 2022-12-16

**Authors:** Pratibha Rao(Lahiri), Sumana Panja, MunMun Chattopadhyay, Jyotirmoy Biswas, Arkadeep Dhali, Gopal Krishna Dhali

**Affiliations:** 1 Department of Physiology, North Bengal Medical College, Siliguri, IND; 2 Department of Physiology, Medical College Kolkata, Kolkata, IND; 3 Department of General Medicine, College of Medicine and Sagore Dutta Hospital, Kolkata, IND; 4 Department of Gastrointestinal Surgery, School of Digestive and Liver Diseases, Institute of Postgraduate Medical Education and Research, Kolkata, IND; 5 Department of Gastroenterology, School of Digestive and Liver Diseases, Institute of Postgraduate Medical Education and Research, Kolkata, IND

**Keywords:** covid-19 pandemic, medical curriculum, medical education, online learning, medical students

## Abstract

Background

In 2020 WHO declared the coronavirus disease 2019 (COVID-19) outbreak as a global pandemic. To flatten the curve of infection, a nationwide lockdown was declared by the Indian government. All the schools and colleges were shut for an indefinite period. Like all other streams, medical education also got severely hampered. Adapting themselves to the changing environment, teachers started using different teaching-learning methods and media to get across to the students. The objective of the research was to study the perception of medical students towards online teaching during the COVID pandemic.

Methods

A descriptive cross-sectional study was conducted by the distribution of a pre-validated online questionnaire to medical students of West Bengal. From the collected data, relevant statistical averages and census domains were calculated. The chi-square test was done and assessed with a p≤0.05 significance level.

Results

A significant increase was noted in the time spent by students on various online teaching activities (p<0.05). Video tutorials, e.g., YouTube, were ranked as the most effective mode (17.2%), followed by live tutorials via Microsoft Teams, etc. (8.9%). A significant number (30.2%) of students strongly favoured online teaching over face-to-face teaching. Major challenges of online learning cited by students were internet connectivity issues (79.8%) followed by family distraction (37.9 %) and inconvenient timing of the classes (20.1%).

Conclusion

Our study highlighted the benefits, disadvantages and barriers to online learning from the perspective of undergraduate medical education in India. Even though the online mode of teaching was found to be beneficial in the context of the COVID-19 pandemic, it cannot be used as an absolute substitution for face-to-face teaching in the given context.

## Introduction

Human coronavirus 2019, also known as severe acute respiratory syndrome coronavirus 2 (SARS-CoV-2) [1], is the virus that causes COVID-19 (coronavirus disease 2019), the respiratory illness. The World Health Organization declared the outbreak a Public Health Emergency of International Concern on 30 January 2020, and a pandemic on 11 March 2020, and human-to-human transmission of SARS-CoV-2 was confirmed on 20 January 2020, during the COVID-19 pandemic [2,3]. Hence to limit the spread of infection simple measures such as physical distancing, wearing a mask, washing hands frequently, keeping rooms well ventilated and avoiding crowds were advised.

Further, in an attempt to flatten the curve of infection, a nationwide lockdown was declared by the Indian government. For that very reason, all the schools and colleges including medical colleges were shut indefinitely. Due to this, medical education like all other streams of education got severely hampered. Once COVID-19 was declared a pandemic, National Medical Commission (NMC) approved the conduction of online classes in medical colleges and clarified that online teaching would be considered valid for the current pandemic period only [4]. After this declaration by NMC, all medical colleges across the country shifted to an online mode of teaching, and all of a sudden, this unprecedented COVID-19 pandemic resulted in an explosion in the adoption of various forms of online teaching methodology. Adapting themselves to the changed environment, teachers started using different teaching-learning methods and media to get across to the students. Some colleges used technology to take live classes through different platforms such as Microsoft Teams, Zoom, Google Meet, Google Classroom, etc. Others uploaded their pre-recorded classes on YouTube, while others created WhatsApp groups with their students and uploaded audio-enabled PowerPoint presentations.

India started the implementation of competency-based medical education (CBME) in August 2019. The competency-based medical education (CMBE) favours skill development and focuses on the production of competent Indian Medical Graduates who are globally relevant and can meet society’s increasing health needs. The undergraduate medical education programme is designed with the goal to create an ‘Indian Medical Graduateʼ (IMG) possessing requisite knowledge, skills, attitudes, values and responsiveness, so that they may function appropriately and effectively as a physician of first contact of the community while being globally relevant to cater to health needs of the society. The institution has to provide a platform that enables the medical student to achieve individual goals and those in the nation’s interests and at the same time cater to the student’s needs to become a compassionate leader, a communicator, a member of the health care team, a lifelong learner and a professional [5].

Due to this pandemic, for the past two years, online teaching has played a key role in medical education and even today remains an important teaching and learning methodology in medical institutes [6]. The sudden transition from on-campus learning to exclusively distance learning is challenging for both faculty and students and has required a lot of preparation and other efforts in a short time [7]. The faculty have risen to the occasion and trained and equipped themselves to meet the demands of the current situation. The younger generation, though more technologically adept, has also had to face its share of challenges to adapt to this novel way of teaching and learning. It is very challenging to create a sense of social presence in distance learning and students feel isolated and do not feel a part of the learning community. It is difficult to assess the level of student learning and to regularly communicate with them without being face-to-face [8]. This transformation has a great impact on the students’ learning.

Challenges to online education reported in the medical literature so far include issues relating to time management, the use of technology tools, students' assessment, communication, and the lack of in-person interaction [9]. Besides, online education may not be equitable in terms of its access and the quality of teaching. Some students do not have access to laptops, smartphones or high-speed internet at home and they are technophobic too [10]. Thus, as online education is growing continuously in its usage with popularity and accessibility, we decided to do a study to find out the perception of medical students towards online teaching during the COVID pandemic along with the perceived benefits and barriers of online teaching.

## Materials and methods

This is a descriptive cross-sectional study. An online survey, using Google Forms was conducted on MBBS students of Phases 1 and 2 of selected medical colleges in West Bengal. The questionnaire consisted of 20 pre-validated questions. Questions were prepared based on current online teaching methods being adopted in different medical colleges of this state and the effects of COVID-19 on medical education. Questions exploring the experiences of online teaching were based on sections I to IV of the Dundee Ready Education Environment Measure (DREEM), a validated questionnaire designed to measure the educational environment of medical colleges and healthcare professionals [11]. These were 5-point Likert-type questions, ranging from strongly disagree to strongly agree. The remaining items in the questionnaire comprised a mixture of question styles [12,13]. Our questions tried to find the general demographics of students, students' use and experience of online teaching, students’ perception of online learning, the role of online learning on practical teaching, and perceived barriers to online teaching.

Participants

All undergraduate medical students from Phase 1 and Phase 2 of four (two Urban & two Rural) NMC-recognised medical colleges were requested to participate. The study was carried out in two steps. In the first step, consent was taken from the students and in the second step, responses were collected from the consenting students using Google Forms. Prior to seeking consent, the students were sensitized to the entire process, and it was clarified to them that all data collected would remain anonymous and would be used for research purposes only. They were free to discuss with friends and family before consenting to be a part of this online survey. 

The Google Form was then distributed to all the consenting volunteers. Here they had to mandatorily click on a box indicating their consent at the very beginning of the form. The Google Form was so designed to allow one-time response only. The process of data collection began only after receiving the institutional Ethical Clearance. Data was collected over a span of one month, from June 2021 to July 2021. 

Data analysis

Data thus collected was exported from Google response to Microsoft Excel. Multiple responses were accounted for in the statistical calculation. All statistical computations were performed using IBM SPSS, version 20 (IBM Corp, Armonk, NY).

## Results

We conducted a cross-sectional observational survey, in which a total of 827 students participated (Table 1).

**Table 1:** Academic year and demography of students

Year of study	Female, n(%)	Male, n(%)	Prefer not to say, n(%)
First	246(39.5)	371(59.6)	5(0.8 )
Second	85(41.4)	119(58)	1(0.5)
Grand Total	331(40.1)	490(59.2)	6(0.1)

Prior to the pandemic only 6.8% of students spent more than 14 hours per week on online teaching platforms which increased to 26.5% of students reportedly spending more than 20 hours per week and 16.3% of students spending between 10 to 20 hours per week during the pandemic. Even though the students spent an increased amount of time engaging in online study activities, the majority of them (70.9%) felt that online teaching has not successfully replaced the offline mode of teaching and learning. Video tutorials e.g., YouTube was ranked as being the most effective method of online learning. Other methods considered were Live tutorials via Zoom/similar platforms, online question banks and digital flashcards (Table 2).

**Table 2:** Method of online learning found to be the most effective presently

Responses (1= Most effective, 5= Least effective)	Video tutorials e.g. YouTube, n(%)	Live tutorials via Zoom/similar platforms, n(%)	Online question banks, n(%)	Online/Digital Flashcards, n(%)	Other, n(%)	Chi-square	p-value
1	143(17.2)	74(8.9)	58(7)	42(5.1)	59(7.1)	28.3288	0.000011 *
2	153(18.4)	148(17.8)	162(19.6)	136(16.4)	89(10.7)		
3	149(18)	250(30.2)	272(32.8)	240(28.9)	177(21.4)		
4	196(23.6)	202(24.4)	199(24)	238(28.7)	171(20.6)		
5	189(22.8)	155(18.7)	138(16.6)	173(20.9)	333(40.2)		

Table 3 reflects that 20.9% of students strongly felt that online teaching is as effective as face-to-face teaching, 17.6% of students felt strongly that online teaching is often stimulating and 19.6% of the students felt strongly that they could engage themselves in these online learning methods. Also 20% of the students felt strongly that they were comfortable in asking their doubts online and 18.1% of students said they enjoyed this form of teaching-learning very much. Around 33.9% of the students felt that the teachers were well prepared for the teaching sessions while only 5.4% strongly disagreed. Around 18.1% of students felt strongly that they were being well prepared for their profession by the online teaching methodologies while 15.2% felt strongly about the inadequacy of this method. Another significant finding that emerged was that a significant (30.2%)number of students strongly favoured online teaching over face-to-face teaching while 21.6% of the students were strongly against this concept.

**Table 3:** Response of the students towards their experience of online teaching

Grade of Responses (1=strongly disagree, 5=strongly agree)	The teaching is often stimulating, n(%)	I find it easy to engage in the lesson, n(%)	I feel able to ask the questions I want, n(%)	I enjoy online teaching, n(%)	I would like the online teaching to be more interactive, n(%)	I feel that online teaching is as effective as face-face teaching, n(%)	I prefer online teaching to face-face teaching, n(%)	The teachers are well prepared for the teaching sessions, n(%)	I feel I am being well prepared for my profession, n(%)	My internet connection is problematic, n(%)
1	60(7.2)	93(11.2)	86(10.4)	124(14.9)	35(4.2)	191(23)	179(21.6)	45(5.4)	126(15.2)	77(9.3)
2	168(20.3)	184(22.2)	161(19.4)	173(20.9)	90(10.9)	174(21)	115(13.9)	104(12.6)	170(20.5)	145(17.5)
3	282(34)	243(29.3)	219(26.5)	233(28.1)	143(17.2)	158(19.1)	148(17.9)	177(21.3)	223(26.9)	186(22.4)
4	173(20.9)	147(17.7)	196(23.7)	149(18)	204(24.6)	133(16)	136(16.4)	222(26.8)	160(19.3)	165(19.9)
5	146(17.6)	162(19.6)	167(20)	150(18.1)	357(43.1)	173(20.9)	251(30.2)	281(33.9)	150(18.1)	256(30.9)

Reduction in travel time (62%) and ability to learn at once, at their own pace (59.3%), were cited as the major advantages. Other advantages included more comfortable setting, flexibility, the ability to ask questions, interactivity and cost savings. The major challenge faced by students was poor internet connectivity (75.8%) followed by family distractions (46.4%). Other barriers included timing of classes, anxiety, lack of devices and lack of space. Around 46.1% of the students felt that the sessions were sufficiently interactive. When asked about the mode of interactivity, the majority of the students (71.3%) found the opportunity for voice interaction during the teaching session the most helpful. The majority of the students (70.7 %) felt that their institution followed a preset curriculum while according to only 4.1% of students, the learning was based on student requests.

## Discussion

Due to the current pandemic situation, we have been forced to make significant changes in how we approach academic activities. Schools, colleges, and universities have been closed and online education has quickly become the new norm all over the world [14]. To keep the young generation interested and motivated, medical colleges across India had started slowly but steadily incorporating e-learning in different forms alongside traditional learning methods and the pandemic aided in speeding up this process. ‘Electronic (e) or online learning can be defined as the use of electronic technology and media to deliver, support and enhance both learning and teaching and involves communication between learners and teachers utilizing online contentʼ [15].

The pandemic brought about a sudden shift from the traditional offline mode to the online mode of teaching and forced the faculty and students to adjust to this new norm. A recent study showed that medical students wanted to participate and give their opinion regarding plans and policies for online classes and the issues relating to their implementation [16]. Hence, we decided to conduct a survey among the Phase 1 and 2 MBBS students across West Bengal to find out about their perception of online learning. We also wanted to find out if this teaching methodology can be incorporated into medical education soon.

Utilization of online teaching methodologies by students

Our study showed that during the pandemic there was a significant increase in the time spent by students on various online teaching activities (p<0.05). Prior to the pandemic, only 11% of the students invested up to 10 hours a week in online learning activities which increased to 20.2% of students spending up to 10 hours a week on online learning activities. This is comparable to the findings of Dost et al., who in their survey of 2721 UK medical students found that students spent an average of seven to 10 hours using online teaching platforms during the pandemic, compared with four to six hours prior to the pandemic and this difference was statistically significant (p<0.05) [17].

Our survey shows that among the different online modes of learning, Video tutorials, eg., Youtube was the most preferred mode by students (17.2%), followed by Live tutorials (8.9%). These are comparable with a study done in the UK which shows that students found video tutorials, for example, YouTube/Osmosis to be the most effective, followed by online question banks, and live tutorials. Reasons for this may include the short, organized and aesthetic nature of pre-recorded videos [18].

Even though the amount of time the students engaged in online teaching activities showed an increase during the pandemic, our survey revealed that the majority of the students (70.7%) felt that online teaching had not successfully replaced the offline mode of teaching and learning. Another significant finding was that an overall majority (46.6%) of students favoured online teaching over face-to-face teaching while 35.5% of the students were against this concept.

Perception

Thirty-six per cent of students in our survey felt that online teaching is as effective as face-to-face teaching, whereas 23% of students strongly disagreed. This is a slight increase over the findings of a survey conducted by the Medical College of Pakistan, where 23% of participants had a positive perception of online teaching [19] but in contrast to another study conducted at the University of Tasmania, according to which 95% of students had a favourable opinion about online teaching and 75% thought it was effective in increasing their skills [20]. A study conducted by Saurabh et al. showed that more than 50% of their students showed a preference for traditional learning [21].

Our survey showed that the majority of the students did not find the online teaching sessions to be stimulating, engaging or enjoyable. Only 17.6% of the students felt strongly that online teaching was stimulating, 19.6% of the students felt strongly that they could engage themselves in these online learning methods and 18% of students said they enjoyed this form of teaching-learning very much. This resonates well with a study done in the UK in which overall the medical students did not find online teaching to be engaging or enjoyable, with limited opportunities to ask questions (BMJ) as against Poland where a vast majority (73%) of the students rated online classes as enjoyable [17,22].

A large section of the students (54.1%) felt that the online teaching sessions adopted by their institutes were not very interactive while 45.9% of the students felt that the sessions were sufficiently interactive. The majority of the students indicated that they found voice interaction during the teaching session and live quizzes the most helpful. On similar lines, a study done in Poland (Polish Study) found that the students were relatively less active during online classes compared to traditional classes. One of the reasons could be the lack of an interactive approach during the online learning sessions. In contrast, the study done in the UK found that 59.73% of students felt that their online classes were interactive enough with students finding the opportunity to interact via the chat box or by directly speaking to the lecturer. Interactivity can be achieved via student response systems, incorporating methods such as polls, quizzes, or breakout rooms, as these have been shown to encourage student participation. Indeed, previous literature suggests the incorporation of online Q&A sessions to improve student engagement [23].

According to a majority of the students (70.7%), the institutes followed a preset curriculum, whereas only 4.1 % of students felt that it was modified based on students' requests. And 25.2% of students indicated that it was a combination of both. These findings are also in sync with the study done in the UK which found that online teaching in medical schools followed a pre-set curriculum; as stated by 66.12% of students, was designed following student requests (3.38%), or using a combination of both (30.50%). This shows that the faculty tried to take the students' opinion into consideration in the delivery of online teaching in both cases [17].

Even though many of the teachers had to update themselves with the use of modern technology, a majority of the students (60.7%) felt that the teachers were well prepared for the teaching sessions while only 18% felt that they were not. In spite of the relatively high score given to the teachers, the quality of the sessions may have been affected by several factors such as poor internet connection, family distractions and the timing of the tutorials, as revealed by our results. Also, it may always be useful to introduce measures and initiatives focused on faculty training for the establishment of successful online learning programs [15].

On being questioned about the adequacy of online teaching for their professional preparedness, the overall majority either found it inadequate (35.7%) or did not have any definite opinion (26.9%). The loss of immediate feedback may have contributed to this, as generally students and doctors prefer face-to-face sessions for communication and feedback purposes [24].

According to the Polish study, while online classes did enable students to acquire knowledge to an extent similar to traditional learning, but, in their opinion, it is definitely less effective in terms of increasing their clinical and social skills [22]. Only 3.3% of students in our study felt that they were being able to learn practical skills via online teaching, while a majority of 85.9% were against this notion. 

Challenges of online teaching

The major factors which were identified as challenges and barriers to online teaching were poor Internet connectivity, the timing of the classes, family distractions, lack of proper space for online classes, lack of an appropriate device and anxiety. About 75.8% of the students reported poor internet connectivity as the major barrier, followed by family distractions (37.9%). Many of the students felt that the times when the classes were held were not convenient for them. Others reported that they did not own hardware which was able to support online classes. Some of them felt that there was no space in their home where they could attend online classes without distractions. There was a small group who reported that the online classes were a source of anxiety for them.

Dost et al., in their study, found the main barriers to online teaching to be family distractions, Internet connection and the timing of tutorials [17]. Harden found that one of the important challenges to online learning was technological limitations for faculties and students alike [25]. Bediang et al. have mentioned how in a low-income country such as Cameroon, poor internet connectivity, Wi-Fi and access to physical infrastructure and hardware are issues faced during online classes [26].

The prerequisites for online learning are a reliable internet connection and the relevant hardware and software. For online teaching to be successful and productive, students and teachers must be knowledgeable and comfortable with the technicalities. Medical institutes must have a strong IT department with good infrastructure and logistic support.

 Benefits/advantages

The advantages and benefits of online learning are varied. According to the majority of students (61.9%), the major advantage of online learning is that it requires no travelling and as a result, many resources (time, money) are saved. Another major advantage was the ability to learn at one’s own pace by reviewing recorded lectures at convenient times (59.2%). Other benefits include flexibility and cost-effectiveness. Many students felt that online learning was more comfortable for them and allowed them to ask questions more freely and engage in more interactive sessions. According to Mooney and Bligh, learning can enable students to have ‘easier and more effective access to a wider variety and greater quantity of informationʼ [27]. According to Baczek et al., the students felt that ease of access to educational materials and the other miscellaneous benefits include increased convenience due to reduced costs as accommodation and transportation were not necessary for online learning [22]. Another benefit was access to resources regardless of location and time. Online learning allows learning materials to be quickly delivered to students including any updates to the material [28]. One other indirect benefit was the reduction of air pollution, for example, carbon dioxide emission as less transportation was being utilized.

Limitations

Our study had some limitations. The main drawback is that it is a cross-sectional survey study which was done in a specific regional medical teaching institution in India. Even though some studies are done on a similar topic, none are done in the context of Indian socio-economic background. Future studies from different regional representation in India is required to generalise the findings.

Suggestions 

Online classes to teach clinical skills are most effective when combined with traditional classes. Video lectures and tutorials either alone or in combination were found to be better for teaching practical skills compared to only text-based study materials.

Faculty-focused training programmes will go a long way to make the technology associated with online teaching easier to handle for faculty members who may not be proficient. Incorporating more interactive sessions in the online teaching sessions will serve to make the lectures more engaging and will encourage the students to interact and participate more.

## Conclusions

Our study highlighted the benefits, disadvantages and barriers to online learning from the perspective of undergraduate medical education in India. The effectiveness of various modes of online teaching like YouTube video tutorials, Zoom live classes, online question banks, etc. were found to be almost similar. A majority of the students have preferred the online mode of teaching as a substitute for the offline one. In contrast, a considerable number of students have suggested that there was a lack of interaction in online teaching in comparison to offline face-to-face teaching. Even though the online mode of teaching was found to be beneficial in the context of the COVID-19 pandemic, it cannot be used as an absolute substitution for face-to-face teaching in the given context.

## References

[1] Lai CC, Shih TP, Ko WC, Tang HJ, Hsueh PR (2020). Severe acute respiratory syndrome coronavirus 2 (SARS-CoV-2) and coronavirus disease-2019 (COVID-19): the epidemic and the challenges. Int J Antimicrob Agents.

[2] (2020). Statement on the second meeting of the International Health Regulations (2005) Emergency Committee regarding the outbreak of novel coronavirus (2019-nCoV). World Health Organization (WHO) (Press release). 30 January. https://www.who.int/news/item/30-01-2020-statement-on-the-second-meeting-of-the-international-health-regulations-(2005)-emergency-committee-regarding-the-outbreak-of-novel-coronavirus-(2019-ncov).

[3] Chan JF, Yuan S, Kok KH (2020). A familial cluster of pneumonia associated with the 2019 novel coronavirus indicating person-to-person transmission: a study of a family cluster. Lancet.

[4] https://timesofindia.indiatimes.com/india/nmc-online-meded-classes-valid-during-pandemiconly/articleshow/78471257.cms.

[5] (2018). Competency based undergraduate curriculum for the Indian Medical Graduate. Medical Council of India.

[6] Moran J, Briscoe G, Peglow S (2018). Current technology in advancing medical education: perspectives for learning and providing care. Acad Psychiatry.

[7] Li L, Xv Q, Yan J (2020). COVID-19: the need for continuous medical education and training. Lancet Respir Med.

[8] Nimavat N, Singh S, Fichadiya N (2021). Online medical education in India: different challenges and probable solutions in the age of COVID-19. Adv Med Educ Pract.

[9] Esani M (2010). Moving from face-to-face to online teaching. Clin Lab Sci.

[10] Rajab MH, Gazal AM, Alkattan K (2020). Challenges to online medical education during the COVID-19 pandemic. Cureus.

[11] Miles S, Swift L, Leinster SJ (2012). The Dundee Ready Education Environment Measure (DREEM): a review of its adoption and use. Med Teach.

[12] Roff S, McAleer S, Harden RM (1997). Development and validation of the Dundee Ready Education Environment Measure (DREEM). Med Teach.

[13] Morawo A, Sun C, Lowden M (2020). Enhancing engagement during live virtual learning using interactive quizzes. Med Educ.

[14] Evans DJ, Bay BH, Wilson TD, Smith CF, Lachman N, Pawlina W (2020). Going virtual to Support anatomy education: a STOPGAP in the midst of the Covid-19 pandemic. Anat Sci Educ.

[15] O'Doherty D, Dromey M, Lougheed J, Hannigan A, Last J, McGrath D (2018). Barriers and solutions to online learning in medical education: an integrative review. BMC Med Educ.

[16] Rajab MH, Gazal AM, Alkawi M, Kuhail K, Jabri F, Alshehri FA (2020). Eligibility of medical students to serve as principal investigator: an evidence-based approach. Cureus.

[17] Dost S, Hossain A, Shehab M, Abdelwahed A, Al-Nusair L (2020). Perceptions of medical students towards online teaching during the COVID-19 pandemic: a national cross-sectional survey of 2721 UK medical students. BMJ Open.

[18] Guo PJ, Kim J, Rubin R (2014). How video production affects student engagement: an empirical study of MOOC videos. L@S '14.

[19] Abbasi S, Ayoob T, Malik A, Memon SI (2020). Perceptions of students regarding E-learning during Covid-19 at a private medical college. Pak J Med Sci.

[20] Warnecke E, Pearson S (2011). Medical students' perceptions of using e-learning to enhance the acquisition of consulting skills. Australas Med J.

[21] Saurabh MK, Patel T, Bhabhor P, Patel P, Kumar S (2021). Students' perception on online teaching and learning during COVID-19 pandemic in medical education. Maedica (Bucur).

[22] Bączek M, Zagańczyk-Bączek M, Szpringer M, Jaroszyński A, Wożakowska-Kapłon B (2021). Students' perception of online learning during the COVID-19 pandemic: a survey study of Polish medical students. Medicine (Baltimore).

[23] Muir S, Tirlea L, Elphinstone B (2020). Promoting classroom engagement through the use of an online student response system: a mixed methods analysis. J. Stat. Educ.

[24] Paechter M, Maier B (2010). Online or face-to-face? Students’ experiences and preferences in e-learning. Internet High Educ.

[25] Harden RM (2006). Trends and the future of postgraduate medical education. Emerg Med J.

[26] Bediang G, Stoll B, Geissbuhler A, Klohn AM, Stuckelberger A, Nko'o S, Chastonay P (2013). Computer literacy and E-learning perception in Cameroon: the case of Yaounde Faculty of Medicine and Biomedical Sciences. BMC Med Educ.

[27] Mooney GA, Bligh JG (1997). Information technology in medical education: current and future applications. Postgrad Med J.

[28] Zehry K, Halder N, Theodosiou L (2011). E-Learning in medical education in the United Kingdom. Procedia Soc Behav Sci.

